# Increased levels of prolactin receptor expression correlate with the early onset of lupus symptoms and increased numbers of transitional-1 B cells after prolactin treatment

**DOI:** 10.1186/1471-2172-13-11

**Published:** 2012-03-09

**Authors:** Yadira Ledesma-Soto, Francisco Blanco-Favela, Ezequiel M Fuentes-Pananá, Emiliano Tesoro-Cruz, Rafael Hernández-González, Lourdes Arriaga-Pizano, María V Legorreta-Haquet, Eduardo Montoya-Diaz, Luis Chávez-Sánchez, María E Castro-Mussot, Adriana K Chávez-Rueda

**Affiliations:** 1 Unidad de Investigación Médica en Inmunología, Hospital de Pediatría, CMN Siglo XXI, IMSS. Av Cuauhtemoc 330. Col. Doctores, Mexico, D.F. CP06720, Mexico; 2 Unidad de Investigación Médica en Enfermedades Infecciosas y Parasitarias, Hospital de Pediatría, CMN Siglo XXI, IMSS. Av Cuauhtemoc 330. Col. Doctores, Mexico, D.F., Mexico; 3 Departamento de Investigación Experimental y Bioterio del Instituto Nacional de Ciencias Médicas y Nutrición "Salvador Zubirán", Vasco de Quiroga 15, Col Tlalpan, Mexico, D.F., Mexico; 4 Unidad de Investigación Médica en Inmunoquímica, Hospital de Especialidades, CMN Siglo XXI, IMSS. Av Cuauhtemoc 330. Col. Doctores, Mexico, D.F., Mexico; 5 Departamento de Inmunología, Escuela Nacional de Ciencias Biológicas, IPN. Prolongación de Carpio y Plan de Ayala s/n Col. Santo Tomas., Mexico D.F., Mexico

## Abstract

**Background:**

Prolactin is secreted from the pituitary gland and other organs, as well as by cells such as lymphocytes. Prolactin has an immunostimulatory effect and is associated with autoimmune diseases that are characterised by abnormal B cell activation, such as systemic lupus erythematosus (SLE). Our aim was to determine if different splenic B cell subsets express the prolactin receptor and if the presence of prolactin influences these B cell subsets and correlates with development of lupus.

**Results:**

Using real-time PCR and flow cytometry, we found that different subsets of immature (transitional) and mature (follicular, marginal zone) B cells express different levels of the prolactin receptor and are differentially affected by hyperprolactinaemia. We found that transitional B cells express the prolactin receptor at higher levels compared to mature B cells in C57BL/6 mice and the lupus-prone MRL/lpr and MRL mouse strains. Transitional-1 (T1) B cells showed a higher level of prolactin receptor expression in both MRL/lpr and MRL mice compared to C57BL/6 mice. Hyperprolactinaemia was induced using metoclopramide, which resulted in the development of early symptoms of SLE. We found that T1 B cells are the main targets of prolactin and that prolactin augments the absolute number of T1 B cells, which reflects the finding that this B cell subpopulation expresses the highest level of the prolactin receptor.

**Conclusions:**

We found that all B cell subsets express the prolactin receptor but that transitional B cells showed the highest prolactin receptor expression levels. Hyperprolactinaemia in mice susceptible to lupus accelerated the disease and increased the absolute numbers of T1 and T3 B cells but not of mature B cells, suggesting a primary effect of prolactin on the early stages of B cell maturation in the spleen and a role of prolactin in B cell differentiation, contributing to SLE onset.

## Background

Prolactin (PRL) is a lactogenic hormone that is mainly produced by the anterior pituitary gland. PRL has multiple functions that regulate reproduction, development and growth, osmosis, metabolism of carbohydrates and lipids and the immune system. Each of these functions requires expression of the PRL receptor in different extra-pituitary regions [1]. In the immune system, interaction between hormones and receptors activates the transcription of genes involved in different cellular functions, such as proliferation, differentiation, and cytokine production [2-4]. PRL has been implicated as a modulator of both cellular and humoral immunity [1-4].

Elevated serum levels of PRL have been reported in several autoimmune diseases, including multiple sclerosis [5] and systemic lupus erythematosus (SLE) [6-9], although this finding has not been reported for other diseases such as autoimmunity during chronic hepatitis C [10]. Moreover, women with hyperprolactinaemia but without autoimmune disorders have been reported to have circulating autoantibodies [11].

SLE is an autoimmune rheumatic disease. Serum samples from SLE patients characteristically have very strong reactivity to a broad spectrum of nuclear components, including DNA, RNA, histones, RNP, Ro, and La. These antibodies form immune complexes that are deposited in the kidneys and may cause proteinuria and kidney failure. The presence of these autoantibodies indicates abnormalities in the activation and development of B cells [12,13], and both B and T cells express the PRL receptor and produce and secrete PRL [1,14-16]. SLE mainly affects women of the reproductive age at a ratio of 9:1 compared to men, and this gender bias has been attributed to the immunostimulatory properties of hormones. SLE symptoms tend to start or become exacerbated during pregnancy, when serum PRL levels are high. High serum concentrations of PRL correlate with SLE activity [6-8], and hyperprolactinaemic patients with antiphospholipid syndrome display significantly more serositis and peritonitis compared to healthy individuals. [9,17]. These findings have also been observed in the murine NZB × NZW model of lupus after the induction of hyperprolactinaemia, in which the presence of PRL correlates with the early detection of immune complexes, proteinuria, and accelerated death [18].

MRL-MpJFas^lpr ^(MRL/lpr) mice have a mutation in the Fas gene and develop a disease similar to SLE, characterised by glomerulonephritis, vasculitis, splenomegaly, hypergammaglobulinemia and the production of anti-dsDNA antibodies [19]. In this strain of mouse, eliminating B cells using an anti-CD79 antibody decreased manifestations of the SLE-like disease, demonstrating the importance of B cells in SLE physiopathology [20,21]. B cells start their maturation process in the bone marrow, undergoing the proB, preB and immature stages, and finish maturation in the spleen, where the transitional and mature B cell subsets can be found. These populations are distinguished by the expression of different surface molecules. Allman et al. classified transitional B cells into three types: transitional-1 (T1 [CD93^+^, IgM^high^, CD23^-^]), transitional-2 (T2 [CD93^+^, IgM^high^, CD23^+^]), and transitional-3 (T3 [CD93^+^, IgM^low^, CD23^+^]), while mature B cells are classified as follicular (FO [CD93^-^, CD21^int^, CD23^high^]) or marginal zone (MZ [CD93^-^, CD21^high^, CD23^-^]) [22].

The objective of this study was to determine whether different splenic B cell subsets express the PRL receptor and if the presence of PRL influences these B cells subsets and correlates with the development of lupus. We found that all B cell subsets expressed the PRL receptor but that transitional B cells displayed higher expression levels compared to mature B cells. Hyperprolactinaemia in mice susceptible to lupus accelerated the disease and increased the absolute numbers of T1 and T3 B cells but not mature B cells, suggesting that PRL participates in the early stages of splenic B cell development.

## Results

### Expression of the PRL receptor varies among the different splenic B cell subsets

**Figure 1:**
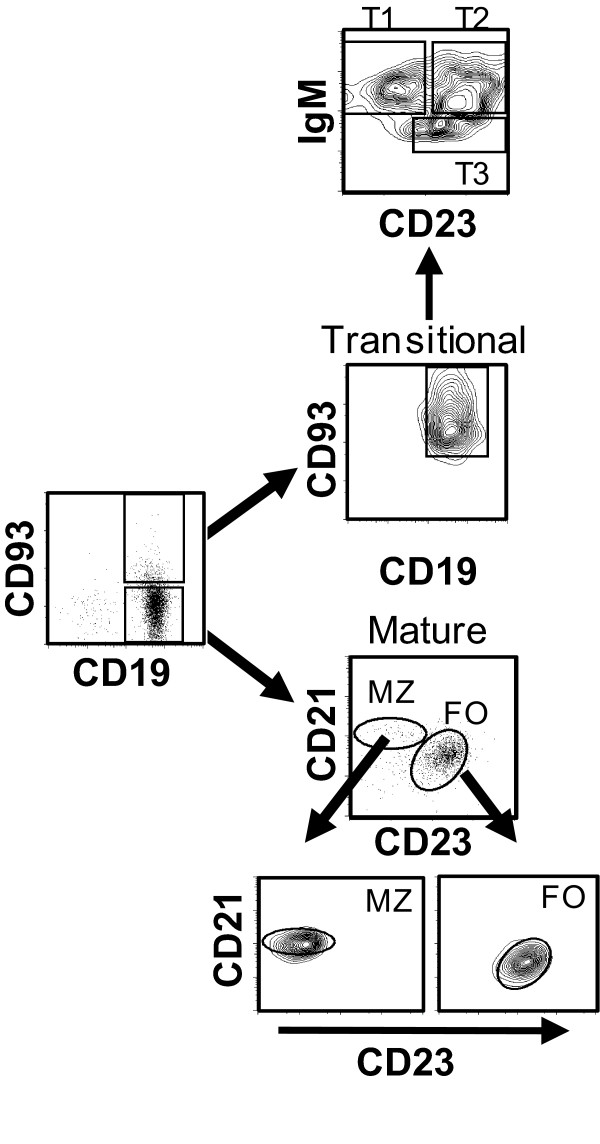
**Purification of B cell subsets by flow cytometry**. B cells were purified by negative selection from 9-week-old mice. Splenic cells were incubated with different antibodies (anti-CD19, anti-CD93, anti-CD21, anti-CD23, and anti-IgM), and the different B cell subsets were purified by flow cytometry as detailed in the Methods section. The purity of the collected populations oscillated between 95% and 98%. A representative example of the purified B cells from wild-type C57BL/6 mice is shown.

**Figure 2:**
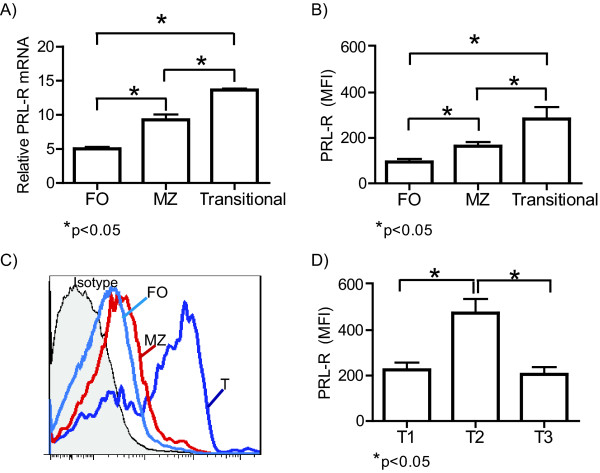
**Expression of prolactin receptor in different subsets of splenic B cells from C57BL/6 mice**. The different B cell subsets from 9-week-old C57BL/6 mice were purified by flow cytometry in four independent experiments using eight mice per experiment. Using real-time PCR, PRL receptor (PRL-R) mRNA expression was determined in the following different subsets of splenic B cells: A) Follicular (FO), marginal zone (MZ), and transitional (T). The protein expression levels of the PRL receptor were determined for ten mice of each strain by flow cytometry, and the mean fluorescence intensity (MFI) is shown in the following B cell subsets: B) Follicular; marginal zone; and transitional. C) Histograms of PRL receptor expression in the different subsets of B cells are shown. D) Transitional 1, 2 and 3. The asterisks denote statistical significance with the p value shown.

The follicular, marginal zone, and transitional splenic B cell subsets were purified from C57BL/6 wild-type mice by flow cytometry with greater than 95% purity (Figure 1) and were assayed for the expression of the PRL receptor at the mRNA and protein levels. We found that splenic B cells expressed the PRL receptor, with transitional B cells showing the highest relative mRNA expression (13.6 ± 0.2). Among the mature B cells subsets, MZ B cells showed approximately two-fold higher expression (9.3 ± 0.8) than the FO B cells (5 ± 0.3) (Figure 2A). At the protein level, we found that transitional B cells expressed the highest level of the PRL receptor (283.3 ± 52.4 MFI [mean fluorescence intensity]), followed by MZ B cells (173.0 ± 18.6 MFI) and FO B cells (103.0 ± 16.0 MFI), similar to the pattern observed with PRL receptor mRNA expression (Figure 2B and 2C). Within the transitional subset, T2 B cells showed the highest protein levels of the PRL receptor (458.7 ± 92.5), followed by T1 (225.2 ± 32.3) and T3 (208.7 ± 59.4) B cells (Figure 2D).

**Figure 3:**
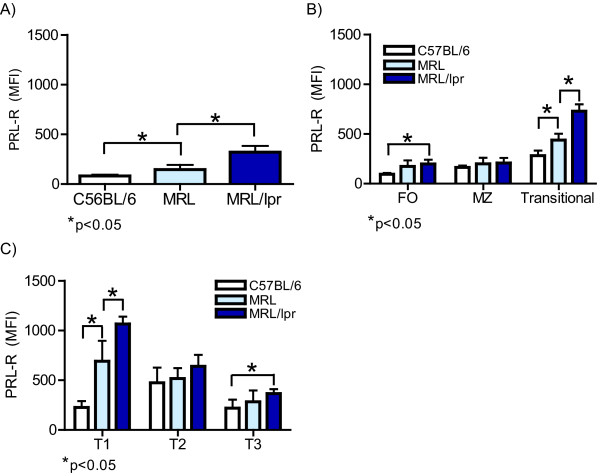
**Expression of prolactin receptor in C57BL/6 wild-type, MRL and MRL/lpr lupus-prone mice**. Protein expression of the PRL receptor (PRL-R) was determined by flow cytometry (MFI) from 14 C57BL/6 and MRL/lpr mice and 7 MRL mice. Splenocytes were marked with anti-CD19, anti-CD93, anti-IgM, anti-CD21, anti-CD23, and goat anti-PRL receptor A) splenic B cells; B) Follicular (FO), marginal zone (MZ), transitional B cells; and C) Transitional 1, 2 and 3. Asterisks denote statistical significance with the p value shown.

When the protein level of PRL receptor among total splenic B cells from C57BL/6 wild-type mice was compared with that in the lupus-prone MRL and MRL/lpr mice, we found a statistically significant (p < 0.05) increase in PRL receptor expression in the MRL mice (two-fold increase; 237.0 ± 93.8 MFI) and in the MRL/lpr mice (three-fold increase; 306.3 ± 58.1 MFI) than in the C57BL/6 mice (96.7 ± 16.6 MFI). The differences in PRL receptor expression between MRL and MRL/lpr mice were also statistically significant (p < 0.05) (Figure 3A).

When individual B cell subsets were evaluated, we observed a statistically significant increase (p < 0.05) in PRL receptor protein expression in FO B cells from MRL/lpr mice (238.0 ± 63.5 MFI) compared to that in cells from C57BL/6 mice (111.2 ± 39.0 MFI). PRL receptor expression in MRL mice was only slightly elevated compared to that in C57BL/6 mice. In MZ B cells, there was not a statistically significant difference in PRL receptor protein expression between C57BL/6, MRL and MRL/lpr mice (190.5 ± 17.6, 201.4 ± 59.1 and 266.0 ± 71.9 MFI, respectively). A statistically significant difference in receptor expression was observed in transitional B cells, with MFI of 805.5 ± 64.6 in the MRL/lpr strain, 437.5 ± 82.6 in MRL strain and 283.5 ± 41.3 in the C57BL/6 mice (Figure 3B). Among the transitional B cell subsets, T1 cells displayed the greatest levels of PRL receptor protein expression in MRL/lpr (1105.7 ± 79.9 MFI) and MRL (749.0 ± 78.0 MFI) mice but not in C57BL/6 (225.5 ± 32.3 MFI) mice. We found similar levels of PRL receptor expression in T2 cells from MRL/lpr (640.0 ± 59.0 MFI), MRL (551.3 ± 75.5 MFI) and C57BL/6 (482.8 ± 159.0 MFI) mice, while T3 cells from the MRL/lpr mice showed the highest level (365.8 ± 43.7 MFI), followed by MRL (310.4 ± 56.8 MFI) and C57BL/6 mice (206.6 ± 94.3 MFI). Of all the splenic B cell subsets, T1 cells from the MRL/lpr strain displayed the highest expression of the PRL receptor (Figure 3C).

### Induction of hyperprolactinaemia results in an early onset of SLE in the disease-prone MRL/lpr and MRL mice and correlates with the increased expression of the PRL receptor in transitional B cells

**Figure 4:**
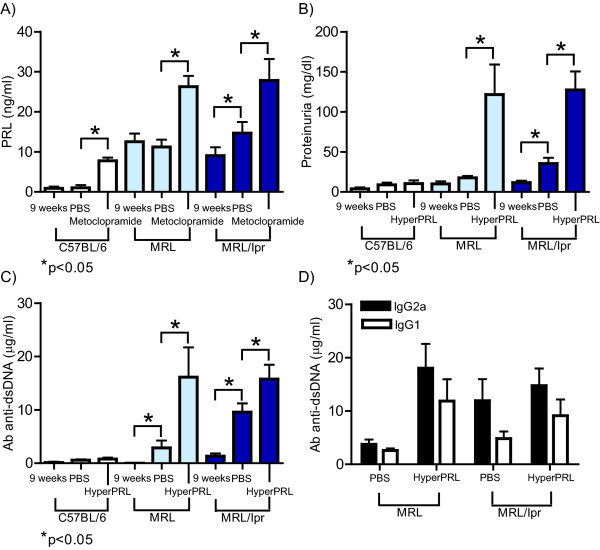
**SLE manifestations in mice with hyperprolactinaemia**. Fourteen C57BL/6 and MRL/lpr mice, and seven MRL mice at 9 weeks of age were treated for 6 weeks with metoclopramide (100 μg/100 μl) or PBS (100 μl). At the end of the treatment, the following measurements were taken: A) PRL concentration by ELISA; B) level of proteinuria using reactive strips; C) anti-dsDNA IgG antibodies; and D) anti-dsDNA antibodies of the IgG1 and IgG2a isotypes in the MRL and MLR/lpr mice by ELISA. The asterisks denote statistical significance between groups with the p value shown. HyperPRL = Hyperprolactinaemic

Nine-week-old MRL/lpr, MRL and C57BL/6 mice were treated with metoclopramide for six weeks to induce high levels of PRL in the serum before testing for accelerated SLE symptoms. The serum concentration of PRL in the C56BL/6 strain was 0.8 ± 0.4 ng/ml before treatment and 1.4 ± 0.9 ng/ml when treated with PBS, while mice treated with metoclopramide showed PRL concentrations that were 10-fold higher than those before treatment (8.1 ± 0.9 ng/ml). In the MRL mice, the serum PRL concentration was 12.5 ± 1.9 ng/ml before treatment, 11.2 ± 1.8 ng/ml when treated with PBS, and 26.2 ± 2.7 ng/ml after being treated with metoclopramide. In MRL/lpr mice, the serum PRL concentration was 9.1 ± 2.4 ng/ml, and this concentration increased to 15.4 ± 2.7 ng/ml in mice treated with PBS and to 29.6 ± 5.3 ng/ml in mice treated with metoclopramide. Although an increase in the PRL concentration was observed in the MRL/lpr mice treated with PBS (mice at 15 weeks of age), this increase did not reach the levels observed in animals treated with metoclopramide (Figure 4A). The consistent increase in the serum concentration of PRL in metoclopramide-treated mice was considered to constitute hyperprolactinaemia and correlated with the premature onset of disease symptoms in lupus-prone mice (see below).

Lupus manifestations were compared in all mouse strains before and after metoclopramide and PBS treatments. The MRL/lpr mice treated with PBS developed proteinuria (50.8 ± 15.7 mg/dl), constituting a significant increase compared to the beginning of the treatment (12.5 ± 4.6 mg/dl). MRL/lpr mice treated with metoclopramide showed a greater increase in proteinuria (104.3 ± 30.7 mg/dl). By comparison, MRL mice developed proteinuria after metoclopramide treatment (118.5 ± 37.4 mg/dl) but not PBS treatment. Metoclopramide-treated, PBS-treated and untreated mice were tested for serum concentrations of anti-dsDNA antibodies of the IgG isotype. A significant increase in anti-dsDNA antibodies was observed in hyperprolactinaemic MRL/lpr mice (16.0 ± 2.9 μg/ml) compared to mice treated with PBS (9.5 ± 1.6 μg/ml) and the levels found at the beginning of the treatment (1.3 ± 0.5 μg/ml). At the beginning of treatment in MRL mice, we did not detect the presence of anti-dsDNA antibodies. However, we observed low concentrations of anti-dsDNA antibodies after PBS treatment (3.2 ± 1.8 μg/ml), and these concentrations increased significantly in hyperprolactinaemic mice (15.1 ± 5.6 μg/ml). The C57BL/6 mice did not develop proteinuria after PBS or metoclopramide treatment, and we did not detect anti-dsDNA antibodies under any condition studied (Figure 4B and C). The main antibody isotypes found in both lupus-prone strains were IgG2a and, to a lesser degree, IgG1 (Figure 4D). No statistically significant differences were found in the production of anti-dsDNA antibodies of the IgM isotype (data not shown). Taken together, these data indicate that metoclopramide treatment induces hyperprolactinaemia and that this state was associated with early manifestations of SLE in the lupus-prone MRL and MRL/lpr mice.

**Figure 5:**
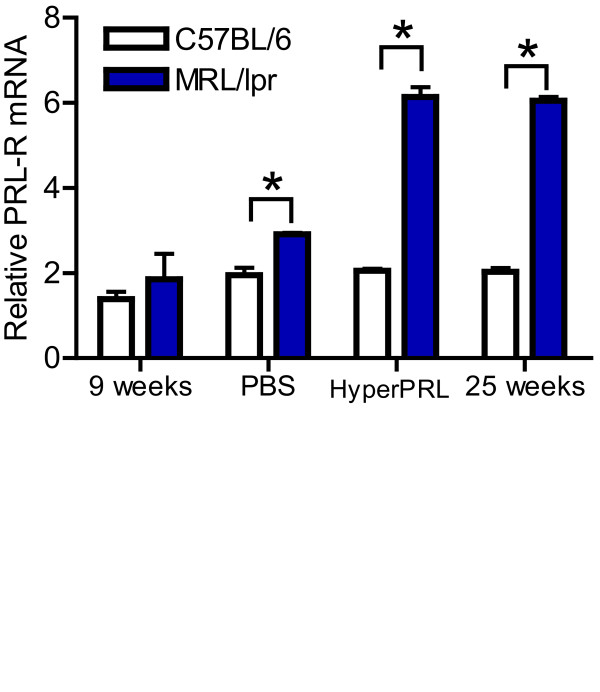
**Prolactin receptor expression in MRL/lpr mice at different ages**. Ten C57BL/6 and ten MRL/lpr mice were treated with metoclopramide (100 μg/100 μl) for 6 weeks [hyperprolactinaemia (HyperPRL)] or PBS (control). Splenic B cells were purified using negative selection from treated and untreated mice: at 9 and 25 weeks of age (untreated) and 15 weeks of age, mice were treated with metoclopramide or PBS (expression of the PRL receptor was determined by real-time PCR [mRNA]). The asterisks denote statistical significance between populations with the p value shown.

Next, we tested PRL receptor expression in whole splenic B cell populations from the hyperprolactinaemic and PBS-treated control mice. However, we did not find any differences in PRL receptor expression in C57BL/6 mice regardless of age or treatment. In contrast, we found that MRL/lpr mice with hyperprolactinaemia showed a significant increased by more than two-fold in PRL receptor expression compared to mice treated with PBS. Therefore, hyperprolactinaemia correlated with upregulation of the PRL receptor in splenic B cell populations. Interestingly, in the MRL/lpr lupus-prone mice, the normal manifestations of SLE occur at 25 weeks of age. When we compared PRL receptor expression in splenic B cells from hyperprolactinaemic mice (15 weeks of age) and untreated diseased mice (25 weeks of age), no differences were found. We found no differences in PRL receptor expression between the C57BL/6 and MRL/lpr strains at 9 weeks of age. In contrast, there was a statistically significant increase in PRL receptor expression in MRL/lpr mice at 15 and 25 weeks of age (p < 0.05) (Figure 5).

**Figure 6:**
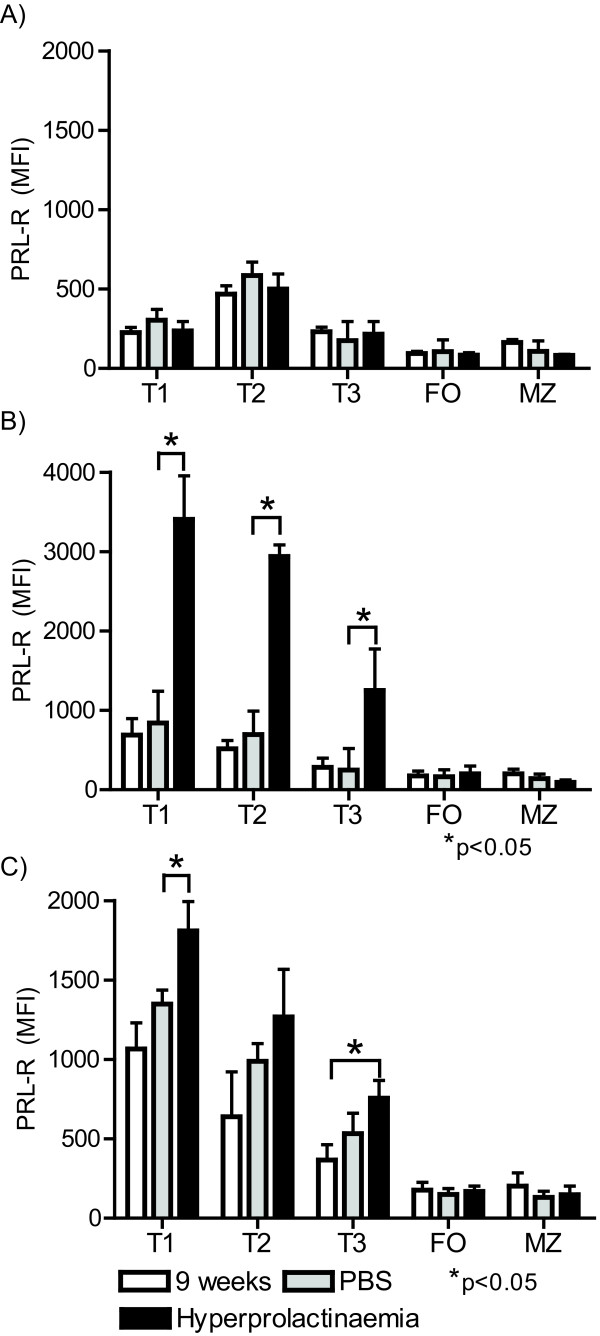
**Prolactin receptor expression after the induction of hyperprolactinaemia**. The levels of PRL receptor protein in transitional (T1, T2, and T3) and mature (FO and MZ) B cells were determined by flow cytometry (MFI). At the end of the treatment, spleen cells were marked with anti-CD19, anti-CD93, anti-IgM, anti-CD21, anti-CD23, and goat anti-PRL receptor. A) C57BL/6 mice; B) MRL mice and C) MRL/lpr mice. The asterisks denote statistical significance between populations with the p value shown.

During hyperprolactinaemia, there was a significant increase in PRL receptor expression in MRL/lpr and MRL mice but not in C56BL/6 mice (Figure 6A). In addition, correlating with the expression patterns of the PRL receptor in non-hyperprolactinaemic conditions (Figure 3), transitional B cells seemed to be the main cells responding to the high levels of PRL by further increasing expression of the PRL receptor. Of the transitional B cells, T1 B cells were the population with the highest increase in PRL receptor expression levels in MRL/lpr [1105.7 ± 79.9 (9 weeks) vs. 1650.2 ± 232.4 MFI (hyperprolactinaemia)] and MRL mice (749.0 ± 78.0 vs. 3407.2 ± 273.2 MFI), although all transitional B cell populations displayed increased PRL receptor expression in the hyperprolactinaemic state in MRL/lpr [T2 (640.0 ± 59.0 vs. 1266.8 ± 300.9 MFI) and T3 (365.8 ± 43.7 vs. 756.0 ± 249.7 MFI)] and MRL [T2 (551.3 ± 75.5 vs. 2938.6 ± 145.1 MFI) and T3 (310.4 ± 56.8 vs. 1251.6 ± 521.6 MFI)] mice (Figures 6B, C). No significant changes were observed in the mature splenic B cell populations. These data suggest a role for PRL in B cell development, mainly in splenic transitional B cells, that correlates with premature manifestations of SLE.

### MZ, but not FO, B cells are altered in untreated and hyperprolactinaemic MRL/lpr lupus-prone mice

**Figure 7:**
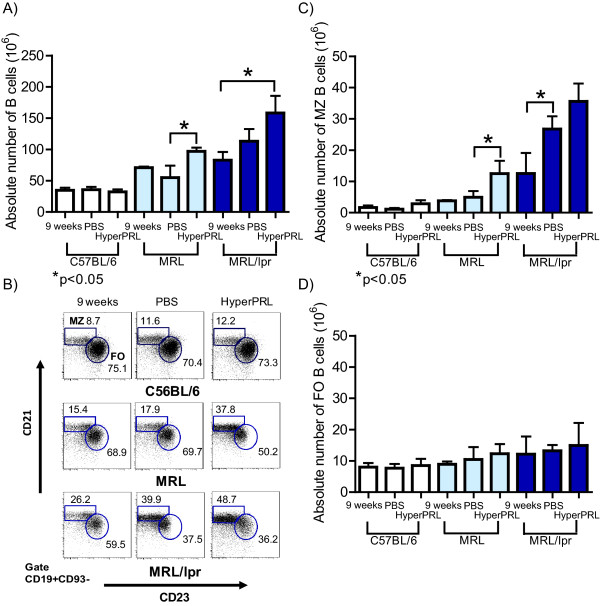
**Absolute numbers of mature B cells after the induction of hyperprolactinaemia**. Nine-week-old mice were treated with metoclopramide (100 μg/100 μl) [hyperprolactinaemia (HyperPRL)] or with PBS (100 μl) for 6 weeks, with fourteen mice per condition in the C57BL/6 and MRL/lpr groups, and seven mice per condition in the MRL group. At the end of this treatment, splenocytes were marked with antibodies against CD19, CD93, CD21, IgM and CD23, and the absolute number of each population was calculated. A) The absolute number of splenic B cells. B) Representative dot plot of the mature MZ and FO B cell subsets. C) Graph of the absolute number of MZ B cells. D) Graph of the absolute number of FO B cells. The asterisks denote statistical significance between populations with the p value shown.

Given that the greatest level of PRL receptor expression was found in immature developing splenic B cells, we evaluated whether PRL influences the absolute number of these cells. In agreement with a role for PRL in developing splenic B cells, we found that hyperprolactinaemic MRL/lpr mice showed an increase in the absolute number of B cells (158.2 ± 27.2 × 10^6 ^cells) compared to untreated mice (82.7 ± 13.19 × 10^6 ^cells). However, no significant difference was found in mice treated with PBS at 15 weeks of age (113.0 ± 29.7 × 10^6 ^cells). Similarly, hyperprolactinaemic MRL mice showed an increase in the absolute number of B cells (104.6 ± 21.3 × 10^6 ^cells) compared to untreated mice (69.9 ± 4.7 × 10^6 ^cells) and PBS-treated mice (59.8 ± 19.1 × 10^6 ^cells) (Figure 7A). Due to the different levels of PRL receptor expression in the different splenic B cells populations, we analysed each B cell subset individually. Figure 7B shows the mature MZ and FO B cell pools before and after metoclopramide and PBS treatment in wild type C57BL/6, MRL and MRL/lpr mice. When these mature B cell subsets were quantified, MZ B cells in MRL/lpr mice showed an increase in absolute numbers that correlated with the development of disease 15 weeks of age compared to 9 week mice (12.5 ± 6.5 × 10^6 ^and 26.7 ± 4.0 × 10^6 ^cells, respectively), but no further significant increases were found in MZ B cells from mice with hyperprolactinaemia (35.5 ± 16.2 × 10^6 ^cells). In MRL mice, we did not observe any differences between mice 9 weeks and those treated with PBS; however, hyperprolactinaemic mice showed an increase in the absolute number of MZ cells (Figure 7C). No differences in FO B cell numbers were detected regardless of the mouse strain or treatment tested (Figure 7D). Similarly, we did not detect any changes in the absolute numbers of FO or MZ B cells from C57BL/6 mice (Figures 7C, and 7D).

### Prolactin preferentially targets transitional-1 B cells and increases their absolute numbers in the spleen

**Figure 8:**
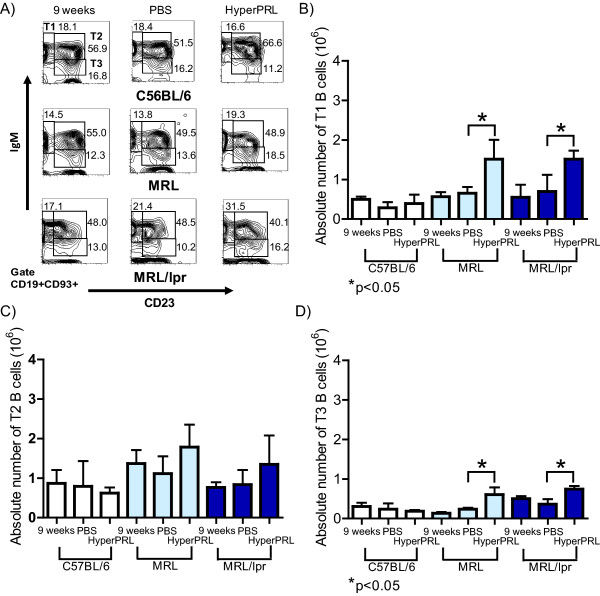
**Absolute number of immature B cells after the induction of hyperprolactinaemia**. Nine-week-old mice were treated with metoclopramide (100 μg/100 μl) [hyperprolactinaemia (HyperPRL)] or PBS (100 μl) for 6 weeks, with fourteen mice per condition in the C57BL/6 and MRL/lpr groups, and seven mice per condition in the MRL group. At the end of the treatment, splenocytes were marked with antibodies against CD19, CD93, CD23, CD21 and IgM. A) Representative dot plot of the transitional B cell subset. B) Graph of the absolute numbers of transitional-1 B cells (T1). C) Graph of the absolute numbers of transitional-2 B cells (T2). D) Graph of the absolute numbers of transitional-3 B cells (T3). The asterisks denote statistical significance between populations with the p value shown.

The largest difference in the absolute number of splenic B cell was found in T1 B cells, correlating with the high level of PRL receptor expression observed in this population. Figure 8A displays the transitional populations and Figure 8B displays the greater than two-fold increase in the absolute number of T1 B cells in the hyperprolactinaemic MRL/lpr mice compared to PBS-treated mice (1.5 ± 0.2 × 10^6 ^cells and 0.7 ± 0.4 × 10^6 ^cells, respectively). The same phenotype was observed in MRL mice, in which the absolute number of T1 B cells only increased in the hyperprolactinaemic mice [1.3 ± 0.1 × 10^6 ^cells (hyperprolactinaemic) and 0.6 ± 0.10 × 10^6 ^cells (PBS)]. In contrast, the absolute number of T2 B cells was not affected by hyperprolactinaemia in any of the strains, as shown in Figure 8C. Moreover, a small but significant increase was found in T3 B cells (Figure 8D) from hyperprolactinaemic MRL/lpr mice (0.8 ± 0.3 × 10^6 ^cells) and MRL mice (0.6 ± 0.1 × 10^6 ^cells) compared to that in cells from PBS-treated mice [MRL/lpr (0.4 ± 0.1 × 10^6 ^cells) and MRL (0.3 ± 0.1 × 10^6 ^cells)]. As expected, there were no differences in the absolute numbers of T1, T2 or T3 B cells between the different treatment groups in C57BL/6 mice. Taken together, these data support a role for PRL in B cell development through the regulation of the size of the T1 B cell pool in the spleen.

## Discussion

Several studies have demonstrated a role for prolactin (PRL) and B cells in the development of autoimmune diseases such as systemic lupus erythematosus (SLE) [6-9,12,13,18,20,21,23,24]. In this study, we evaluated how PRL affects the course of SLE development in MRL/lpr, MRL, and wild-type mice and observed how this finding correlates with changes in the different splenic B cell subsets. The MRL/lpr strain has a mutation in the Fas gene and develops a disease similar to SLE [25]. MRL mice also exhibit autoimmune disorders despite carrying a normal Fas gene, but the symptoms manifest much later in life than in MRL/lpr mice [19]. To our knowledge, this study is the first to address PRL receptor expression in all of the different subsets of splenic B cells.

Interestingly, expression of the PRL receptor followed a similar pattern in B cells from wild-type C57BL/6, SLE-prone MRL/lpr and MRL mice. Expression of the PRL receptor varied according to the B cell developmental stage. The highest expression of the PRL receptor was observed in the immature, transitional splenic B cells. Our findings are consistent with those reported by Morales et al. [26], who transfected proB cells with the PRL receptor triggered their progression to the preB stage through incubation with PRL. Taken together, data from Morales et al. and our group suggest that PRL participates in early B cell differentiation. Our results showed that the pattern of PRL receptor expression levels in transitional B cells was different in C56BL/6 mice and the lupus-prone mouse strains. T2 cells showed higher expression of the PRL receptor in C7BL/6, while in lupus-prone strains the T1 cells showed higher expression. These data argue that there is altered expression of the PRL receptor in different B cells subsets during the autoimmune process. Furthermore, the differential presence of the receptor in transitional populations in C56BL/6 mice suggests a regulatory role for PRL in these cells under non-pathological conditions.

Transitional B cells are constantly testing their antigen receptors (BCR) to identify B cell clones expressing receptors with self-specificity [27]. These clones then trigger different intrinsic mechanisms that eliminate this self-specificity. The MRL strains have a genetic background prone to develop a disease similar to SLE either earlier in life in the case of MRL/lpr mice or later in the case of MRL mice. We found that the T1 B cell subset in both of these strains had the most PRL receptor protein expression. Therefore, it is possible that increased PRL receptor expression at this stage in lupus-prone strains could promote both the rescue of autoreactive clones and B cell developmental progression, thus favouring the development of lupus symptoms. This observation is in agreement with the fact that PRL receptor signalling increases the expression of the anti-apoptotic gene Bcl-2 [28,29] and that T1 B cells from hyperprolactinaemic ovariectomised BALB/c mice are more resistant to apoptosis than those from PBS-treated mice [30]. Future experiments should address the molecular role of PRL in this population and better define its contribution in autoimmune disease.

C57BL/6, MRL and MRL/lpr mice treated with metoclopramide showed a further increase in their serum PRL levels that was only accompanied by increased levels of anti-dsDNA antibodies and proteinuria in the MRL/lpr and MRL lupus-prone strains. These findings are in agreement with previous reports studying other hyperprolactinaemic and SLE models, such as reports by McMurray et al. [18,31] who showed hyperprolactinaemia induced by pituitary gland transplantation in NZB × NZW mice, and a report by Peeva et al. using recombinant PRL to induce hyperprolactinaemia in Sle3/5 R4A-γ2b C57BL/6 mice [32]. Additionally, our data are consistent with several clinical trials showing that a high serum PRL level correlated with SLE disease activity [7,33,34].

In our study, PRL receptor mRNA expression was increased in metoclopramide-treated 15-week-old MRL/lpr mice to a level similar to that found in untreated 25-week-old mice displaying high disease activity. This correlation between PRL receptor levels and the degree of disease suggests that PRL plays an important role in the development and exacerbation of SLE. These results also support the idea that the PRL receptor could be useful as an SLE prognostic marker.

Although our results showed that the T2 subset of B cells express the highest levels of the PRL receptor in the spleens of C57BL/6 mice, the absolute number of T2 cell and other B cell subsets and overall PRL receptor expression were not affected by hyperprolactinaemia, and SLE was not induced. This result differs from previous reports by Peeva et al. and Saha et al. describing decreased T1 B cell frequencies and increased T2 B cell frequencies after the induction of hyperprolactinaemia, while the MZ and FO mature B cell pools were unaffected [30,35]. This finding may be due to the different experimental approaches used, in that different mouse strain was used (BALB/c), BALB/c mice were ovariectomised, a procedure that eliminates oestrogen and progesterone and affects immune responses [36,37]. In addition, we treated mice with metoclopramide to induce the hyperprolactinaemic state, whereas Peeva and Saha used ovine PRL.

In our study, hyperprolactinaemia resulted in up-regulation of PRL receptor expression and a significant increase in the absolute numbers of T1 B cells in the MRL/lpr mice. PBS-treated mice did not show increases in the absolute number of T1 cells, despite an increase in PRL receptor expression, we believe that this is because the level of PRL receptor expression in PBS-treated mice never reached the levels found in either pharmacologically induced or age-related hyperprolactinaemic mice. Additionally, in the MRL strain, the absolute number of T1 cells only increased in hyperprolactinaemic mice, correlating with the early onset of lupus symptoms and increased PRL receptor expression. A similar observation has been noted in NK cell lines, in which high PRL receptor expression correlates with an enhanced capacity of the cells to proliferate [38]. This finding is also in agreement with the observation that the T1 population expresses the highest level of PRL receptor expression before treatment and with other studies showing this subset to be more resistant to apoptosis in mice with hyperprolactinemia [30]. Thus, our data and that of others [30,38,39] support the importance of PRL in B cell development, in developmental associated processes such as proliferation and resistance to negative selection and in the progression of SLE. These findings highlight the idea that this disease originates at different levels, as indicated by its multifaceted nature.

We also found that hyperprolactinaemia increased the expression of the PRL receptor and, to a lesser degree, the absolute numbers of T3 B cells. T3 B cells are considered by Merrel to be a subset of anergic B cells produced by the interaction of the BCR and self-antigens and are not a part of the maturation pathway of B cells [40,41]. The T3 B cell subset is decreased in MRL/lpr mice, and this decrease has been proposed to be due to self-reactive clones escaping from an anergic state in this mouse strain. This diminished T3 population was described in 9-week-old mice; however, this time point is prior to disease onset, and these mice were compared to the genetically unrelated BALB/c strain [42]. Therefore, future work should determine the effect, if any, of PRL signalling in this population and may generate interesting results.

The number of MZ B cells in MRL/lpr mice increased with age and coincided with the course of the disease, as previously reported [43]. Higher levels of serum PRL induced a further increase in the MZ B cell population, although this change was not significant when compared to mice of the same age treated with PBS. However, this increase in the absolute number of MZ cells was statistically significant in hyperprolactinaemic-MRL mice. These results are consistent with a model in which PRL increases the expression of its receptor mainly in immature transitional B cells, leading to self-reactive cell maturation with a potential bias toward the MZ type.

Although PRL did not affect the absolute number of mature B cells or their PRL receptor expression pattern, self-reactive clones were more active in hyperprolactinaemic MRL/lpr and MRL mice as shown by an increased concentration of anti-dsDNA antibodies, specifically of the IgG isotype. It will be interesting to discern the function of PRL in the MZ and FO mature populations, as it is clear that they perform different functions. While MZ B cells are part of the innate immune system and preferentially respond to T cell-independent antigens, FO B cells perform the classic functions of adaptive immunity. MZ B cells have mainly been associated with the expression of IgM antibodies [44,45].

Our results show that both MRL/lpr and MRL mice develop an early onset of lupus symptoms after induction of hyperprolactinemia. Although the MRL/lpr strain has an additional mutation in Fas [19,46], our results also suggest that this genetic change most likely has little, if any, effect on the accelerated lupus development. It is possible that strong PRL signalling in immature T1 B cells from both mice strains could potentially trigger rescue from apoptosis induced by recognition of self-antigens and could also shape and promote their differentiation into specific mature B cell populations. The presence of self-reactive clones in MZ B cells correlates with autoimmune diseases [47].

## Conclusions

Our data support a role for PRL in B cell development by primarily affecting the size of the T1 B cell pool in the spleen. Although PRL exhibits pleiotropic effects in different organs, our results are consistent with a preferential effect in the regulation of the PRL receptor in the T1 population that is responsible for the observed altered B cell development and premature onset of SLE symptoms. Understanding the molecular mechanisms resulting in the development of SLE will help to design prevention, treatment and follow-up strategies for this disease.

## Methods

### Mice

All studies were approved by the Animal Care Committee of Instituto Nacional de Ciencias Médicas y Nutrición "Salvador Zubiran" and Hospital de Pediatría, Centro Médico Nacional Siglo XXI IMSS, and all of the measurements taken from the mice were in accordance with approved guidelines established by Mexico (Norma Oficial Mexicana NOM-062-ZOO-1999) and the NIH Guide for the Care and Use of Laboratory Animals. The C57BL/6 mice were purchased from Harlan (Indianapolis, USA), and the MRL/MpJ FAS^lpr ^(MRL/lpr) and MRL/MpJ (MRL) mice were purchased from the Jackson Laboratory (Maine, USA). All of the mice were housed in a specific pathogen-free barrier facility and were provided sterile food and water *ad libitum*.

### Antibodies

The following antibodies were used: anti-mouse CD21-FITC (7 G6) and CD21-APC (7 G6) were from BD Biosciences (Mountain View CA, USA); CD93-PE (AA4.1), CD23-biotinylated, IgM-APC (11/41), CD19-Cy7 (1D3), CD23-PE-Cy7 (B3B4), CD19-FITC (eBioD3), and IgM-biotinylated (11/41) were from eBioscience (San Diego CA, USA); goat anti-mouse PRL-R (E20) was from Santa Cruz Biotechnology (Santa Cruz CA, USA); and swine anti-goat-biotinylated was from Invitrogen (Carlsbad CA, USA). The biotinylated secondary antibody was detected with streptavidin-phycoerythrin-Cy5.5 from BD Biosciences (Mountain View CA, USA).

### Purification of B cells

Single-cell suspensions were prepared from spleens. After red blood cell lysis with lysing buffer (Sigma Aldrich, St. Louis Missouri, USA), the cells were incubated with anti-CD43 (Ly-48) microbeads (Miltenyi Biotec, Bergisch Gladbach, Germany) and the B cells were isolated using negative selection with a magnetic activated cell-sorting (MACS) system (Miltenyi Biotec, Bergisch Gladbach, Germany). After depletion, > 98% of the remaining cells were CD19^+ ^by flow cytometry.

### Cell sorting

Single-cell suspensions of B cells were incubated with fluorescently labelled antibodies specific for CD19, CD93, CD21, CD23, and IgM in staining buffer (PBS with 0.5% BSA) for 20 minutes at 4°C. The cells were washed, and the B cell (CD19^+^) subsets were isolated according to the expression of the following surface markers: marginal zone (CD93^-^, CD21^high^, CD23^-^); follicular (CD93^-^, CD21^int^, CD23^high^); transitional-1 (CD93^+^, IgM^high^, CD23^-^); transitional-2 (CD93^+^, IgM^high^, CD23^+^); and transitional-3 (CD93^+^, IgM^low^, CD23^+^). Cell sorting was performed using a FACSAria sorter with FACSDiva software (BD Bioscience). The purity of the sorted cells ranged from 95% to 98%.

### Real-time PCR

Total RNA was extracted from B cells of C57BL/6 and MRL/lpr mice using TRIzol reagent (Invitrogen, Carlsbad CA, USA), according to the manufacturer's protocol, and the RNA concentration was determined using UV spectrophotometry. Next, 1 μg of total RNA was used to generate cDNA with SuperScript II reverse transcriptase (Invitrogen, Carlsbad CA, USA), according to the manufacturer's specifications. The PRL receptor cDNA was amplified by real-time PCR using a LightCycler TaqMan Master kit (Roche Diagnostic, Mannheim, Germany), according to manufacturer's specifications and using hydrolysis probes and primers designed by Roche Diagnostic. The following primers were used: PRL-R 5'-CAGTAAATGCCACGAACGAA-3' (left); PRL-R 5'-GAGGAGGCTCTGGTTCAACA-3' (right); β-actin 5'-AAGGCCAACCGTGAAAAGAT-3' (left); and β-actin 5'-GTGGTACGACCAGAGGCATAC-3' (right). The final volume of the reaction was 10 μl, and a LightCycler instrument was used to perform the PCR reaction (Roche Diagnostic). The following PCR conditions were used: 10 minutes at 95°C, followed by 40 cycles of 10 seconds at 95°C, 30 seconds at 60°C, and 1 second at 72°C and 1 cycle cooled for 30 seconds at 40°C. The samples were normalised to the β-actin gene. The relative expression of the PRL receptor was calculated using the 2ΔCT formula.

### Induction of hyperprolactinaemia

Groups of 14 female, 9-week-old C57BL/6 and MRL/lpr mice and 7 female MRL mice were given a daily subcutaneous injection of 100 μg of metoclopramide (Sigma Aldrich, St Louis MO, USA) in 100 μl of PBS for six weeks. A matched control group (C57BL/6, MRL and MRL/lpr) received only PBS (100 μl) over the same period. Urinary protein levels were assessed semiquantitatively using reagent strips for urinalysis (Uri-Quick Stanbio Laboratory, Kendall TX, USA). Serum samples were obtained at the beginning and at the end of the experiments, between 08:00 and 11:00 hours, and kept at -35°C until assayed for prolactin and anti-DNA antibodies.

### Prolactin assessment

Serum levels of prolactin were detected using ELISA. The 96-well maxisorp plates (Nunc, Rochester NY, USA) were coated overnight with 100 μl of 4 μg/ml anti-mouse prolactin monoclonal antibody (clone 207518, R&D Systems, Minneapolis MN, USA) in PBS at 4°C, blocked with 2% bovine serum albumin (Invitrogen, Carlsbad CA, USA), and incubated with the serum sample (1:10) overnight at 4°C. Recombinant mouse prolactin (National Hormone and Peptide Program, NIH, donated by AF Parlow) was used as a standard. The plates were then incubated with 0.1 μg/ml biotinylated anti-prolactin antibody (R & D Systems, Minneapolis MN, USA), avidin-alkaline phosphatase (Zymed Laboratories, San Francisco CA, USA) and 5-bromo-4-chloro-3 indolyl phosphate (Sigma-Aldrich, St Louis MO, USA) as a substrate, according to the manufacturer's instructions. The OD was monitored at 405 nm using a Dynatech MR5000 ELISA reader.

### Determination of anti-DNA antibodies

Anti-dsDNA antibody serum concentrations were detected using ELISA. A 96-well maxisorp plate (Nunc, Rochester NY, USA) was coated with 100 μl of 2.5 μg/ml calf thymus dsDNA (Sigma Aldrich, St Louis MO, USA) in bicarbonate buffer overnight at 4°C and was blocked with 2% bovine serum albumin (Invitrogen, Carlsbad CA, USA). The plates were then incubated for 1 h at 37°C with serum (1:50) or the anti-dsDNA antibody standard (clone 16-13, Chemicon International, Billerica MA, USA), followed by rabbit anti-mouse IgG, IgG1, IgG2a or anti-mouse IgM conjugated to alkaline phosphatase (Zymed Laboratories, San Francisco CA, USA) and substrate ([5-bromo-4-chloro-3- indolyl phosphate; Sigma-Aldrich, St Louis MO, USA]). The OD was monitored at 405 nm using a Dynatech MR5000 ELISA reader.

### Cell surface staining and flow cytometry

Splenocytes were incubated with fluorescently labelled antibodies for 20 minutes at 4°C in staining buffer (PBS with 0.5% BSA and 0.01% sodium azide). The cells were then washed and fixed in 2% paraformaldehyde (Sigma Aldrich, St Louis MO, USA). The data were acquired using a FACSAria flow cytometer (BD Bioscience) and analysed with FlowJo software (Tree Star, Ashland OR, USA).

### Statistical analysis

The data were analysed with standard statistical tests (mean value, SD, Student's *t *test, and ANOVA), and the results are expressed as the mean ± SD. The level of significance was set at p ≤ 0.05. All calculations were performed using SPSS 15 software.

## Competing interests

The authors declare that they have no competing interests.

## Authors' contributions

YLS, MVLH, EMD and LCH performed and analysed the experiments, FBF and EMFP interpreted the results and prepared the manuscript preparation, ETC and RHG performed the mouse experiments, LAP performed the cell sorting, AKCR contributed to the experimental design, interpretation of results, and manuscript preparation. All of the authors read and approved the final manuscript.
